# Research Visibility in Physical Therapy: A Narrative Analysis of Academic and Clinical Contributions

**DOI:** 10.12688/f1000research.180139.1

**Published:** 2026-05-06

**Authors:** Vivek Ramanandi, Aparna Bachkaniwala

**Affiliations:** 1 School of Rehabilitation & Medical Sciences, College of Health Sciences, University of Nizwa, Nizwa, Ad Dakhiliyah ‍Governorate, 616, Oman; 2 Department of Neurological Physiotherapy, SPB Physiotherapy College,, Veer Narmad South Gujarat University, Surat, Gujarat, 395005, India

**Keywords:** Physical therapy; physiotherapy; bibliometrics; evidence-based practice; research visibility; clinician; academic faculty; research barriers.

## Abstract

**Objectives:**

This narrative review examines differences in research visibility between academic and clinical physical therapists, explores structural barriers that limit research engagement in clinical settings, and considers the contribution of practice-based inquiry to the profession’s evidence base.

**Methods:**

A narrative synthesis was undertaken using published literature on scholarly productivity, bibliometric performance, research participation, evidence-based practice, and research barriers among physical therapy academics and clinicians across multiple international contexts. Relevant papers were identified from major health and bibliographic databases and by screening reference lists of eligible articles.

**Results:**

Academic physical therapists consistently demonstrate higher measurable bibliometric output than clinicians, although performance varies across institution type, region, and research infrastructure. In contrast, clinicians across several countries report recurring barriers to research engagement, including heavy workload, limited protected time, inadequate research training, and insufficient institutional support. Despite lower publication visibility, practice-based clinical inquiry generates contextually relevant, patient-centred knowledge that supports the implementation of evidence and service improvement. Clinician-scholars also serve an important bridging role between knowledge generation and clinical application.

**Conclusion:**

Research visibility in physical therapy is shaped by structural conditions rather than scholarly interest alone. Conventional bibliometric measures privilege academic environments and do not adequately capture clinicians’ practice-based contributions. Broader evaluative approaches that recognise clinical audit, quality improvement, and implementation activities are needed to more equitably represent professional contribution and strengthen the discipline’s evidence base.

## Introduction

Research visibility is often assessed using publication counts, citation metrics, and indices such as the h- and g-indices. Across healthcare disciplines, these indicators are often treated as markers of professional value and scholarly influence. In physical therapy, however, such measures tend to favour academic practitioners, whose roles are more closely aligned with research production, publication, and citation accumulation.

Academic physical therapists typically work in environments where research activity is institutionally rewarded through promotion pathways, tenure expectations, accreditation standards, and access to research infrastructure. In contrast, clinical practitioners often work in service-oriented settings characterised by high patient volumes, productivity demands, and limited protected time for scholarship. As a result, the visible outputs commonly used to assess academic contribution may not reflect the full range of professional knowledge generation within the discipline.

This distinction matters for several reasons. First, bibliometric indicators were designed primarily to assess academic productivity and may be less suitable for evaluating clinically embedded scholarship. Second, the growth of physical therapy depends not only on generating new knowledge in academic settings but also on interpreting, adapting, and applying it in routine practice. Third, an increasingly narrow reliance on publication-based metrics risks undervaluing clinicians whose work meaningfully contributes to patient care, quality improvement, and evidence implementation.

This narrative review, therefore, aims to characterise the bibliometric landscape of academic physical therapy, examine structural barriers to research engagement among clinical physiotherapists, and highlight the value of practice-based inquiry as an important yet often under-recognised form of scholarly contribution.

## Methods

A narrative review approach was used to synthesise published literature on research visibility in physical therapy. Electronic databases, including PubMed/MEDLINE, CINAHL, and Scopus, were searched to identify studies on scholarly productivity, bibliometrics, evidence-based practice, research barriers, and research capacity in physical therapy. Reference lists of eligible articles were also examined to identify additional relevant sources.

Studies were considered eligible if they reported bibliometric data on academic physical therapy or Doctor of Physical Therapy faculty, or examined research participation, research barriers, or engagement in evidence-based practice among clinical physiotherapists. Review articles addressing knowledge translation, training programmes, or research capacity in physiotherapy and allied health were also considered if directly relevant to the aims of this paper. No geographic restrictions were applied, allowing comparison across different healthcare and educational systems.

The identified literature was reviewed and thematically organised into three domains: bibliometric output among academic physical therapists, barriers to research engagement among clinical physiotherapists, and the value of practice-based clinical inquiry. As this article is based on a narrative synthesis of published literature, ethical approval was not required.

## Results

### Academic physical therapy: Bibliometric landscape

Published bibliometric studies indicate that academic physical therapists generally have higher measurable scholarly output than clinicians. A national analysis of Doctor of Physical Therapy faculty in the United States reported median values of seven publications, 42 citations, an h-index of 2, a g-index of 5, and an e-index of 5.4. ^1^ These figures offer a broad overview of academic productivity but also mask substantial internal variation.

Regional analyses offer additional perspective. In the south-eastern United States, tenure-track Doctor of Physical Therapy faculty with a promotion had a median of 4 publications, an average of 12.4 citations, and an h-index of 3. ^2^ In the western United States, promoted faculty had a median of three publications, an average of 25.5 citations, and an h-index of 2. ^3^ Earlier work on publication productivity in academic programmes also found wide variation across institutions, with research-intensive universities, such as those classified as Carnegie Research 1 or 2, tending to outperform smaller or less research-focused programmes. ^1, 4^

These differences suggest that bibliometric performance is shaped not only by individual scholarly activity but also by institutional conditions, including access to research infrastructure, mentorship, funding opportunities, doctoral training, protected time, and a supportive academic culture. In this context, high research visibility is partly a function of structural opportunity.

### Clinical physical therapy: Barriers to research engagement

In contrast, the literature consistently shows that clinical physiotherapists face significant barriers to engaging in research. Clinicians often work in environments shaped by heavy patient load, service delivery pressures, limited time, insufficient research training, and restricted access to research resources. These constraints reduce opportunities to conduct formal research even when interest in evidence-based practice is present.

In Kuwait, only a minority of clinical physiotherapists reported active research participation, with lack of time, heavy workload, and limited access to library resources identified as major barriers. ^5, 6^ Similar findings have been reported in Kenya, Ghana, India, Saudi Arabia, Nigeria, and Pakistan, where clinicians commonly describe poor research skills, inadequate institutional support, and difficulty integrating research activity into routine clinical work. ^7– 12^

A systematic scoping review of training programmes aimed at improving evidence uptake among physiotherapists found that barriers to knowledge translation are not solely research-specific but also reflect wider organisational and professional cultural factors. ^13^ This pattern is not limited to physical therapy. Allied health literature similarly indicates that research capacity is strongly influenced by organisational culture, leadership support, mentorship, and access to training. ^14^ Across settings, the main barriers appear structural rather than motivational. Clinicians may value research and evidence-based practice yet remain constrained by the demands and priorities of the environments in which they work.

### Value and impact of clinical research

Although clinicians often have lower bibliometric visibility, practice-based inquiry contributes in important ways to the profession’s evidence base. Clinical audits, outcome monitoring, quality improvement initiatives, and service evaluations frequently address questions that arise directly from patient care. Physiotherapists’ perceptions suggest that engagement increases significantly when research is seen as personally meaningful and directly relevant to practice. ^15^ These forms of inquiry are often highly relevant to real-world decision-making and may generate knowledge that is immediately applicable in local practice settings.

Practice-based evidence has distinct strengths. It reflects the complexity of routine care, captures context-specific service realities, and engages with patient populations that may be underrepresented in tightly controlled research settings. It can therefore complement formal academic research by helping bridge the gap between published evidence and everyday practice.

Clinician-scholars occupy a particularly important position in this process. By working across the boundaries of service and scholarship, they help translate research findings into practical protocols and generate clinically relevant questions for further investigation. Their contribution is central to evidence implementation, yet it is often inadequately represented in publication-focused evaluation systems.

## Discussion

This narrative analysis reveals that the difference in research visibility between academic and clinical physical therapists stems from structural imbalances rather than from varying levels of scholarly dedication. Academic environments reward and enable bibliometric output through institutional mandates, protected time, and research infrastructure; clinical environments do not. Bibliometric indices, designed to measure academic productivity, are therefore inadequate and potentially misleading tools for evaluating clinicians’ professional contributions.

This interpretation is supported by the remarkable consistency of barrier-reporting across culturally distinct settings. ^5– 8, 11, 12^ Across countries from Kuwait to Kenya, Ghana, and Pakistan, the same structural challenges persist, such as high workload, limited time, insufficient research training, and limited institutional support. These are not individual deficiencies to be addressed through motivational interventions; they are systemic features of clinical practice environments that require organisational-level solutions. Interventions demonstrating promise include protected research time, structured mentorship through academic–clinical partnerships, and targeted capacity-building programmes, all of which are validated in both the physiotherapy literature and broader allied health research. ^13, 14^

Furthermore, the current prioritisation of publication-based metrics risks creating a distorted professional taxonomy where academic researchers are systematically valued more than clinical practitioners, regardless of the impact on patient outcomes. This distortion has real-world effects: it can discourage skilled clinicians from choosing practice-focused careers, hinder collaboration among different professionals, and limit the diversity of evidence within the profession. Physical therapy advances by generating new knowledge in academic settings and refining and applying it in clinical practice. Evaluation systems that focus solely on the former overlook the importance of practical application and continuous improvement in clinical settings later.

This review has several limitations. Although the narrative synthesis method is suitable for the review’s scope and aims, it lacks a systematic risk-of-bias evaluation and may be affected by publication bias. Studies identified through database searches might underestimate clinical settings where research culture is still developing or absent, possibly leading to an overestimation of the worldwide prevalence of positive research attitudes. Additionally, comparing the studies directly is challenging due to differences in methods, sample characteristics, and measurement tools. However, the main findings are consistent across diverse healthcare systems and professional groups, supporting the strength of the core conclusions.

## Conclusion

Research visibility in physical therapy is skewed towards academic practitioners primarily because of structural factors, not because of differences in attitude. Bibliometric metrics often fall short in fully capturing the contributions of clinical physiotherapists, as their practice-based inquiry produces contextually important, patient-centred insights. Advancing the profession involves expanding evaluative frameworks to acknowledge all forms of scholarly contributions, such as clinical audit, quality improvement, and evidence implementation. It also requires addressing institutional barriers that hinder clinical research participation. Supporting clinician-led research through dedicated time, mentorship, and organisational backing will strengthen the evidence base in physical therapy and promote equity in a field that depends on both academic rigour and clinical expertise.

## Declaration of AI-assisted language editing

During the preparation of this manuscript, the authors used ChatGPT (OpenAI, paid version) and Grammarly Premium to assist with grammar checking, language refinement, content review, and restructuring for clarity and readability. These tools were used solely to improve the manuscript’s presentation. All suggestions and edits were carefully reviewed, verified, and approved by the authors, and the authors take full responsibility for the final content of the manuscript.

## Data Availability

No new underlying data are associated with this article. This manuscript is based on a narrative synthesis of previously published literature, all of which is cited in the reference list. No extended data are associated with this article.
