# Research Visibility in Physical Therapy: A Narrative Analysis of Academic and Clinical Contributions

**DOI:** 10.12688/f1000research.180139.2

**Published:** 2026-07-10

**Authors:** Vivek Ramanandi, Aparna Bachkaniwala

**Affiliations:** 1 School of Rehabilitation & Medical Sciences, College of Health Sciences, University of Nizwa, Nizwa, Ad Dakhiliyah ‍Governorate, 616, Oman; 2 Department of Neurological Physiotherapy, SPB Physiotherapy College,, Veer Narmad South Gujarat University, Surat, Gujarat, 395005, India

**Keywords:** Physical therapy; physiotherapy; bibliometrics; evidence-based practice; research visibility; clinician; academic faculty; research barriers.

## Abstract

**Objectives:**

This narrative review examines differences in research visibility and professional recognition between academic and clinical physical therapists. It specifically distinguishes among structural barriers to traditional research production, clinicians’ contributions to implementation and evidence translation, and the potential value of clinician-led research within the profession’s evidence base.

**Methods:**

A narrative synthesis was undertaken using published literature on scholarly productivity, bibliometric performance, research participation, evidence-based practice, and research barriers among physical therapy academics and clinicians across multiple international contexts. Relevant papers were identified from major health and bibliographic databases and by screening reference lists of eligible articles.

**Results:**

Academic physical therapists consistently demonstrate higher measurable bibliometric output than clinicians, although performance varies across institution type, region, and research infrastructure. In contrast, clinicians across several countries report recurring barriers to traditional research engagement, including heavy workload, limited protected time, inadequate research training, and insufficient institutional support. Clinicians also perform essential evidence implementation, outcome monitoring, auditing, quality improvement, and service evaluation activities that support patient care and service development. These activities should not be treated as identical to traditional research outputs, but they represent clinically valuable professional contributions that are often under-recognised. Clinician-scholars also serve an important bridging role between knowledge generation and clinical application.

**Conclusion:**

Research visibility in physical therapy depends on structural factors, not just scholarly interest. Traditional bibliometric metrics favour academic settings and overlook clinicians’ practical contributions, such as audits and quality improvement. Broader approaches are necessary to recognise these efforts while distinguishing research productivity, evidence translation, and clinical service.

## Introduction

Research visibility is often assessed using publication counts, citation metrics, and indices such as the h- and g-indices. Across healthcare disciplines, these indicators are often treated as markers of professional value and scholarly influence. In physical therapy, however, such measures tend to favour academic practitioners, whose roles are more closely aligned with research production, publication, and citation accumulation.

Academic physical therapists typically work in environments where research activity is institutionally rewarded through promotion pathways, tenure expectations, accreditation standards, and access to research infrastructure. In contrast, clinical practitioners often work in service-oriented settings characterised by high patient volumes, productivity demands, and limited protected time for scholarship. As a result, the visible outputs commonly used to assess academic contribution may not reflect the full range of knowledge-related professional work within the discipline.

A clearer conceptual distinction is therefore needed. In this paper, research visibility primarily refers to that achieved through conventional scholarly outputs, especially publications and citations. Professional recognition refers more broadly to the acknowledgement of clinically valuable work, including the implementation of evidence, outcome monitoring, service improvement, and practice-based inquiry. These concepts are related, but they are not identical. Clinical audit, quality improvement, and service evaluation can support evidence-informed care without necessarily being equivalent to traditional scholarly research or generalisable knowledge production.

This distinction matters for several reasons. First, bibliometric indicators were designed primarily to assess academic productivity and may be less suitable for evaluating clinically embedded scholarship or implementation-supportive work. Second, the growth of physical therapy depends not only on generating new knowledge in academic settings but also on interpreting, adapting, and applying it in routine practice. Third, an increasingly narrow reliance on publication-based metrics risks undervaluing clinicians whose work meaningfully contributes to patient care, quality improvement, and evidence implementation.

This narrative review, therefore, aims to characterise the bibliometric landscape of academic physical therapy, examine structural barriers that limit clinicians’ ability to conduct traditional research, distinguish evidence translation and service improvement contributions from conventional research outputs, and highlight the value of clinician-led research as an important yet often under-recognised component of professional development.

## Methods

A narrative review approach was used to synthesise published literature on research visibility in physical therapy. Electronic databases, including PubMed/MEDLINE, CINAHL, and Scopus, were searched to identify studies on scholarly productivity, bibliometrics, evidence-based practice, research barriers, and research capacity in physical therapy. Reference lists of eligible articles were also examined to identify additional relevant sources.

Studies were considered eligible if they reported bibliometric data on academic physical therapy or Doctor of Physical Therapy faculty, or examined research participation, research barriers, or engagement in evidence-based practice among clinical physiotherapists. Review articles addressing knowledge translation, training programmes, or research capacity in physiotherapy and allied health were also considered if directly relevant to the aims of this paper. No geographic restrictions were applied, allowing comparison across different healthcare and educational systems.

The identified literature was reviewed and thematically organised into three domains: bibliometric output among academic physical therapists, barriers to research engagement among clinical physiotherapists, and the value of practice-based clinical inquiry and evidence implementation. As this article is based on a narrative synthesis of published literature, ethical approval was not required.

## Results

### Academic physical therapy and traditional research production: Bibliometric landscape

Published bibliometric studies indicate that academic physical therapists generally have higher measurable scholarly output than clinicians. A national analysis of Doctor of Physical Therapy faculty in the United States reported median values of seven publications, 42 citations, an h-index of 2, a g-index of 5, and an e-index of 5.4. ^1^ These figures offer a broad overview of academic productivity but also mask substantial internal variation.

Regional analyses offer an additional perspective. In the south-eastern United States, tenure-track Doctor of Physical Therapy faculty with a promotion had a median of 4 publications, an average of 12.4 citations, and an h-index of 3. ^2^ In the western United States, promoted faculty had a median of three publications, an average of 25.5 citations, and an h-index of 2. ^3^ Earlier work on publication productivity in academic programmes also found wide variation across institutions, with research-intensive universities, such as those classified as Carnegie Research 1 or 2, tending to outperform smaller or less research-focused programmes. ^1, 4^

These differences suggest that bibliometric performance is shaped not only by individual scholarly activity but also by institutional conditions, including access to research infrastructure, mentorship, funding opportunities, doctoral training, protected time, and a supportive academic culture. In this context, high research visibility is partly a function of structural opportunity. The higher visibility of academic physical therapists should therefore be interpreted in relation to the systems that enable traditional research production, rather than as a direct comparison of professional commitment across academic and clinical roles.

### Clinical physical therapy: Structural barriers to traditional research engagement

In contrast, the literature consistently shows that clinical physiotherapists face significant barriers to engaging in traditional research. Clinicians often work in environments characterised by heavy patient loads, service-delivery pressures, limited time, insufficient research training, and restricted access to research resources. These constraints reduce opportunities to conduct formal research even when interest in evidence-based practice is present.

In Kuwait, only a minority of clinical physiotherapists reported active research participation, with lack of time, heavy workload, and limited access to library resources identified as major barriers. ^5, 6^ Similar findings have been reported in Kenya, Ghana, India, Saudi Arabia, Nigeria, and Pakistan, where clinicians commonly describe poor research skills, inadequate institutional support, and difficulty integrating research activity into routine clinical work. ^7– 12^

A systematic scoping review of training programmes aimed at improving evidence uptake among physiotherapists found that barriers to knowledge translation are not solely research-specific but also reflect wider organisational and professional cultural factors. ^13^ This pattern is not limited to physical therapy. Allied health literature similarly indicates that research capacity is strongly influenced by organisational culture, leadership support, mentorship, and access to training. ^14^ Across settings, the main barriers appear structural rather than motivational. Clinicians may value research and evidence-based practice yet remain constrained by the demands and priorities of the environments in which they work.

### Evidence implementation, service improvement, and practice-based inquiry

Although clinicians often have lower bibliometric visibility, practice-based inquiry contributes in important ways to evidence-informed care. Clinical audits, outcome monitoring, quality improvement initiatives, and service evaluations frequently address questions that arise directly from patient care. Physiotherapists’ perceptions suggest that engagement increases significantly when research is seen as personally meaningful and directly relevant to practice. ^15^ These activities are often highly relevant to real-world decision-making and may generate knowledge that is immediately applicable in local practice settings.

At the same time, it is important to avoid conflating all forms of practice-based activity with traditional scholarly research. Audits, quality improvement projects, outcome tracking, and service evaluations are often locally focused and may not be designed to generate findings that are generalisable beyond a particular service or institution. Their value lies partly in supporting the responsible interpretation and implementation of evidence, improving clinical processes, and strengthening service quality. This contribution deserves professional recognition, but it should be recognised through criteria appropriate to its purpose and methodological scope.

Practice-based evidence has distinct strengths. It reflects the complexity of routine care, captures context-specific service realities, and engages with patient populations that may be underrepresented in tightly controlled research settings. It can therefore complement formal academic research by helping bridge the gap between published evidence and everyday practice. Clinician-scholars occupy a particularly important position in this process. By working across the boundaries of service and scholarship, they help translate research findings into practical protocols and generate clinically relevant questions for further investigation. Their contribution is central to evidence implementation, yet it is often inadequately represented in publication-focused evaluation systems.

### Clinician-led research and the academic-clinical interface

Supporting clinician-led research is also important for strengthening the profession. Clinicians are closely engaged with patient priorities, service constraints, and everyday care challenges. As a result, they are often well placed to identify research questions that are meaningful to patients and frontline practitioners. Clinician-led research can therefore complement academic research agendas by grounding inquiry in practical clinical problems and by supporting translation between evidence generation and service delivery.

However, clinician-generated work should not be romanticised. Not all clinician-led projects are methodologically rigorous or broadly impactful, and some audit or quality improvement activities are undertaken because they are feasible within constrained clinical environments rather than because they are designed to advance generalisable knowledge. The professional task is therefore not to collapse the distinction between research productivity and clinical service contribution, but to create pathways through which clinically important questions can be developed into rigorous research where appropriate, while also recognising high-quality implementation and service-improvement work on its own terms.

## Discussion

This narrative analysis suggests that the difference in research visibility between academic and clinical physical therapists stems largely from structural imbalances rather than from differences in scholarly dedication. Academic environments reward and enable bibliometric output through institutional mandates, protected time, mentorship, and research infrastructure; clinical environments often do not. Bibliometric indices, designed to measure academic productivity, are therefore limited and potentially misleading when used as the sole tools for evaluating the professional contributions of clinicians.

The first major implication is that structural barriers limit clinicians’ ability to conduct traditional research. This interpretation is supported by the consistency of barrier-reporting across culturally distinct settings. ^5– 8^^,^^11, 12^ Across countries from Kuwait to Kenya, Ghana, and Pakistan, the same challenges persist, including high workload, limited time, insufficient research training, and limited institutional support. These are not individual deficiencies to be addressed only through motivational interventions; they are systemic features of clinical practice environments that require organisational-level solutions. Interventions demonstrating promise include protected research time, structured mentorship through academic-clinical partnerships, and targeted capacity-building programmes, all of which are supported in both the physiotherapy literature and broader allied health research. ^13, 14^

The second implication is that clinicians’ evidence-translation and implementation work requires more precise recognition. Research investment has limited practical value unless clinicians remain current with the evidence, interpret it appropriately, adapt it to patient and service contexts, and integrate it into care. This work is central to the profession, but it is not always visible in publication counts or citation indices. Recognition could therefore include structured documentation of evidence implementation, audit and quality improvement portfolios, outcome-monitoring systems, contribution to clinical guidelines or protocols, and participation in academic-clinical research partnerships. These indicators should be used carefully, with attention to quality, methodological appropriateness, patient relevance, and service impact, rather than treated as direct substitutes for peer-reviewed publications.

The third implication is that clinician-led research should be actively supported because it can strengthen the relevance and applicability of the physical therapy evidence base. Clinician-researchers can identify questions emerging from patient priorities and frontline service challenges. They can also help translate academic findings into practical protocols and can generate feedback from real-world implementation. At the same time, the value of clinician-led work depends on methodological rigour, ethical conduct, appropriate design, transparent reporting, and meaningful dissemination. A balanced evaluative framework should therefore recognise both academic rigour and clinical relevance without assuming that every practice-based activity has the same purpose or evidentiary status.

Furthermore, the current prioritisation of publication-based metrics risks creating a distorted professional taxonomy in which academic researchers are systematically valued more than clinical practitioners, regardless of their contributions to patient outcomes, service quality, and evidence implementation. This distortion can discourage skilled clinicians from developing practice-focused scholarly identities, hinder collaboration among different professional groups, and limit the diversity of evidence within the profession. Physical therapy advances by generating new knowledge in academic settings and by refining, adapting, and applying that knowledge in clinical practice. Evaluation systems that focus solely on the former overlook the importance of practical application and continuous improvement in clinical settings.

This review has several limitations. Although the narrative synthesis method is suitable for the review’s scope and aims, it lacks a systematic risk-of-bias evaluation and may be affected by publication bias. Studies identified through database searches might underestimate clinical settings where research culture is still developing or absent, possibly leading to an overestimation of the worldwide prevalence of positive research attitudes. Additionally, comparing the studies directly is challenging due to differences in methods, sample characteristics, and measurement tools. However, the main findings are consistent across diverse healthcare systems and professional groups, supporting the strength of the core conclusions.

## Conclusion

Research visibility in physical therapy is skewed towards academic practitioners primarily due to structural factors, not differences in attitude or professional commitment. Bibliometric metrics are useful for describing traditional research productivity, but they are insufficient for capturing clinicians’ contributions to evidence implementation, service improvement, practice-based inquiry, and patient-centred care. Advancing the profession requires clearer distinctions between research production, evidence translation, and clinical service contribution. It also requires broader evaluative frameworks that recognise clinical audit, quality improvement, outcome monitoring, evidence implementation, and clinician-led research in ways that are rigorous, realistic, and appropriate to their purpose. Supporting clinician-led research through dedicated time, mentorship, academic-clinical partnerships, and organisational backing will strengthen the evidence base in physical therapy and promote equity in a field that depends on both academic rigour and clinical expertise.

## Declaration of AI-assisted language editing

During the preparation of this manuscript, the authors used ChatGPT (OpenAI, paid version) and Grammarly Premium to assist with grammar checking, language refinement, content review, and restructuring for clarity and readability. These tools were used solely to improve the manuscript’s presentation. All suggestions and edits were carefully reviewed, verified, and approved by the authors, and the authors take full responsibility for the final content of the manuscript.

## Data Availability

No new underlying data are associated with this article. This manuscript is based on a narrative synthesis of previously published literature, all of which is cited in the reference list. No extended data are associated with this article.
