# Supplementary material for: Research Visibility in Physical Therapy: A Narrative Analysis of Academic and Clinical Contributions
Source: F1000Res. 2026 Jul 10;15:673. Originally published 2026 May 6. [Version 2] doi: 10.12688/f1000research.180139.2 (PMC13555033; doi:10.12688/f1000research.180139.2)
Supplement: Peer review history

# Reviewer report: Reviewer response for version 2

**DOI:** 10.5256/f1000research.205594.r501181

**Published:** 2026-07-24

**Authors:** Hari Babu

**Affiliations:** 1 P P Savani University, Dhamdod, Gujarat, India

This narrative review Provides a clear and comprehensive Summary of research visibility in physical therapy, by contrasting academic bibliometric production with clinicians and contributions to evidence translation.

 The article's conclusions are reasonable and well balanced, and it is written in an understandable style. The manuscript effectively highlights the imbalance between academic and clinical research visibility. To further strengthen its contribution, the authors could go beyond describing these challenges and propose practical frameworks for recognising clinicians scholarly work.

Is the review written in accessible language?

Yes

Are all factual statements correct and adequately supported by citations?

Yes

Are the conclusions drawn appropriate in the context of the current research literature?

Yes

Is the topic of the review discussed comprehensively in the context of the current literature?

Yes

Reviewer Expertise:

Physiotherapy in Musculoskeletal and Cardiorespiratory

I confirm that I have read this submission and believe that I have an appropriate level of expertise to confirm that it is of an acceptable scientific standard.

# Response

**Published:** 2026-07-24

**Authors:** Vivek Ramanandi

**Affiliations:** Faculty of Physiotherapy, Marwadi University, Rajkot, Gujarat, India

Dear Dr Hari Babu

 Thank you for your expert opinion and suggestions for improvement.

 Best Regards.

# Reviewer report: Reviewer response for version 2

**DOI:** 10.5256/f1000research.205594.r500190

**Published:** 2026-07-20

**Authors:** Mark Elkins

**Affiliations:** 1 The University of Sydney, Sydney, New South Wales, Australia

I'm happy with the revisions.

Is the review written in accessible language?

Partly

Are all factual statements correct and adequately supported by citations?

Partly

Are the conclusions drawn appropriate in the context of the current research literature?

Partly

Is the topic of the review discussed comprehensively in the context of the current literature?

Partly

Reviewer Expertise:

I do my own clinical research in respiratory physiotherapy. I mentor clinicians in the public hospital system who are seeking to do research alongside their clinical roles. I do research into the methodological standards of physiotherapy research. I am the editor of the top-ranked physiotheray journal. I co-direct the Physiotherapy Evidence Database.

I confirm that I have read this submission and believe that I have an appropriate level of expertise to confirm that it is of an acceptable scientific standard.

# Response

**Published:** 2026-07-20

**Authors:** Vivek Ramanandi

**Affiliations:** Faculty of Physiotherapy, Marwadi University, Rajkot, Gujarat, India

Dear Reviewer,

 Thank you for your positive report.

 Best Regards.

# Reviewer report: Reviewer response for version 1

**DOI:** 10.5256/f1000research.198725.r486137

**Published:** 2026-06-17

**Authors:** Mark Elkins

**Affiliations:** 1 The University of Sydney, Sydney, New South Wales, Australia

The manuscript addresses an important and under-discussed issue in physical therapy: the structural imbalance between academic and clinical environments in opportunities for research engagement and in recognition of that engagement. The paper is strongest when discussing the institutional barriers faced by clinicians, including lack of protected time, limited infrastructure, and insufficient organisational support for research participation. However, in its current form, the manuscript conflates several distinct concepts that would benefit from clearer separation and more precise terminology.

 I would encourage the authors to separate their argument into three distinct claims.

 1. Structural barriers limit clinicians’ ability to conduct traditional research

 This is, in my opinion, the manuscript’s clearest and most persuasive argument. Academics are typically employed within systems that provide protected research time, mentorship, funding pathways, and promotion structures tied to scholarly output. Clinicians, by contrast, often work in environments dominated by patient throughput and service delivery pressures.

 As a result, clinicians may preferentially engage in lower-resource activities such as audits, quality improvement projects, and service evaluations because these are feasible within real-world clinical constraints. This is an important structural observation and does not require broadening the definition of scholarship to make the point.

 2. Clinicians contribute important implementation and evidence-translation work that currently receives limited professional recognition

 This is a distinct argument from the first and should be framed separately.

 The manuscript currently risks implying that activities such as audits, QI projects, outcome tracking, and evidence implementation are equivalent to traditional scholarly outputs. Many readers will not accept this equivalence because these activities are often locally focused, methodologically limited, and typically do not generate knowledge that is generalisable to other institutions.

 However, there is a strong and defensible argument that clinicians perform critically important implementation-supportive work that is undervalued within existing professional reward structures. Research investment ultimately has limited value unless clinicians:

- remain current with the evidence,
- interpret it appropriately,
- and integrate it into patient care.

This translational and implementation role is essential to the profession and arguably under-recognised.

 That said, the manuscript currently does not operationalise what form this proposed “recognition” should take. Should these activities influence promotion criteria? Credentialing? Professional awards? Institutional KPIs? The paper would benefit from specifying how broader recognition might realistically and rigorously occur without collapsing distinctions between research productivity and high-quality clinical practice.

 3. Supporting clinician-led research is likely to strengthen the profession

 The manuscript would also benefit from more explicitly acknowledging that clinician-led research can make uniquely valuable contributions to the literature.

 Some of the most clinically meaningful research arises from clinician-researchers because they are closely engaged with patient priorities and everyday care challenges. Clinician-researchers are often well positioned to identify questions that matter directly to patients and frontline practitioners. In contrast, academic research agendas may at times be shaped by funding availability, methodological feasibility, or institutional publication pressures rather than immediate clinical relevance.

 At the same time, it is important not to romanticise clinician-generated work. Not all clinician-led projects are rigorous or impactful, and some audits/QI activities are undertaken primarily because they are achievable within constrained environments rather than because they advance knowledge substantially. A more balanced discussion acknowledging both realities would strengthen the manuscript considerably.

 Additional conceptual issue

 I would also encourage the authors to reconsider the term “research visibility.” At several points, the manuscript appears less concerned with visibility of research than with recognition of clinically valuable professional activity. The current terminology sometimes obscures whether the manuscript is arguing:

- that clinicians conduct scholarly work that is insufficiently recognised by bibliometric systems,
  or
- that clinically valuable activities deserve broader professional recognition even when they are not traditionally scholarly.

Clarifying this distinction would improve precision and reduce conceptual ambiguity.

 Overall, I believe the manuscript addresses an important issue and could make a worthwhile contribution if the authors more clearly distinguish between research production, evidence implementation, and clinical service contribution, while also clarifying the specific forms of recognition they propose.

Is the review written in accessible language?

Partly

Are all factual statements correct and adequately supported by citations?

Partly

Are the conclusions drawn appropriate in the context of the current research literature?

Partly

Is the topic of the review discussed comprehensively in the context of the current literature?

Partly

Reviewer Expertise:

I do my own clinical research in respiratory physiotherapy. I mentor clinicians in the public hospital system who are seeking to do research alongside their clinical roles. I do research into the methodological standards of physiotherapy research. I am the editor of the top-ranked physiotheray journal. I co-direct the Physiotherapy Evidence Database.

I confirm that I have read this submission and believe that I have an appropriate level of expertise to state that I do not consider it to be of an acceptable scientific standard, for reasons outlined above.

# Response

**Published:** 2026-06-19

**Authors:** Vivek Ramanandi

**Affiliations:** Faculty of Physiotherapy, Marwadi University, Rajkot, Gujarat, India

Dear Dr Elkins,

 We sincerely thank you for your detailed and constructive review of our manuscript. We appreciate your recognition of the topic's importance and welcome your valuable suggestions to strengthen the article.

 We agree with your suggestions and will revise the manuscript to improve conceptual clarity, terminology, and overall balance. In particular, we will more clearly distinguish between the structural barriers that limit clinician-led traditional research, the important role clinicians play in evidence implementation and quality improvement, and the broader contribution of clinician-led research to the profession. We will also clarify the distinctions among audits, quality improvement initiatives, service evaluations, and scholarly research, while recognising the value of evidence translation activities and the importance of appropriately acknowledging these contributions. In addition, we will refine our use of the term “research visibility” and strengthen the discussion by acknowledging both the benefits of clinician-led research and the need for methodological rigour, generalisability, and meaningful impact. Thank you again for highlighting these important considerations.

 In accordance with the F1000Research review process, we understand that substantial revisions are required for the manuscript to progress. We will carefully consider your recommendations, along with any additional peer review reports received, and prepare a revised version that responds constructively to the issues raised. We are confident that your feedback will help us improve the article's clarity, balance, and overall contribution.

 Thank you once again for your valuable review and for providing guidance that will meaningfully enhance the manuscript's strength and scholarly quality.

 Sincerely,

 Vivek Ramanandi and Aparna Bachkaniwala
